# Long non-coding RNA implicated in the invasion and metastasis of head and neck cancer: possible function and mechanisms

**DOI:** 10.1186/s12943-018-0763-7

**Published:** 2018-01-24

**Authors:** Xiaobo Luo, Yan Qiu, Yuchen Jiang, Fangman Chen, Lu Jiang, Yu Zhou, Hongxia Dan, Xin Zeng, Yu L. Lei, Qianming Chen

**Affiliations:** 1 State Key Laboratory of Oral Diseases & National Clinical Research Center for Oral Diseases & Dept. of Oral Medicine of West China Hospital of Stomatology, Sichuan University, No. 14, Sec.3, Renminnan Road, Chengdu, Sichuan 610041 China; 2 Department of Periodontics and Oral Medicine, University of Michigan School of Dentistry Comprehensive Cancer Center, 1600 Huron Parkway 2355, Ann Arbor, MI 48109 USA; 3 Laboratory of Pathology, Clinical Research Center for Breast, West China Hospital, Sichuan University, Chengdu, 610041 Sichuan China; 4 Department of Pathology, West China Hospital, Sichuan University, Chengdu, 610041 Sichuan China; 5 University of Michigan Comprehensive Cancer Center, Ann Arbor, MI 48109 USA; 6 Department of Otolaryngology-Head and Neck Surgery, University of Michigan Health System, 1600 Huron Parkway 2355, Ann Arbor, MI 48109 USA

**Keywords:** Long noncoding RNA, Invasion, Metastasis, Head and neck cancer, Mechanism

## Abstract

Head and neck cancer (HNC) ranks as the 6th most common malignancy across the world. Metastasis is a hallmark of cancer, primarily contributing to the relapse and poor prognosis of HNC. Recently, long noncoding RNAs (lncRNAs), previously considered as non-functional, are increasingly appreciated by scholars to play crucial roles in mediating HNC metastasis. LncRNAs, which are located in the nucleus and cytoplasm, mainly exert their function via epigenetic modification, transcriptional control and translational regulation. As several lncRNAs are presently demonstrated to participate in HNC metastasis, we make a summary of the functions and mechanisms regarding these lncRNAs. As shown in the literature, most lncRNAs appear to promote the metastasis of HNC. Hence, we primarily discuss the lncRNAs involved in enhancing metastasis. Additionally, more studies are needed to understand those lncRNAs without clear mechanisms. Furthermore, we introduced the upstream regulator for the aberrant expression of lncRNAs in HNC. Finally, we concisely addressed future research prospects of lncRNAs, particularly the interplay between lncRNAs and tumor immunity as well as lncRNA-targeted therapeutic techniques, and we introduced clustered regularly interspaced short palindromic repeats (CRISPR)-Display as a possibly transformative tool to study lncRNAs. Although lncRNA research is still in the initial stage, it holds great promise to be applied as a prognosticator of HNC and a therapeutic target to inhibit HNC metastasis, which could significantly enhance the outcome of HNC patients.

## Background

Head and neck cancer (HNC), which ranks sixth among the frequent malignant neoplasms worldwide, has an estimate of over 500,000 new cases detected annually [1, 2]. The broad definition of HNC includes not only the mucosal epithelial carcinomas of the head and neck area, presenting as and head and neck squamous cell carcinoma (HNSCC) along with nasopharyngeal carcinoma (NPC), but also thyroid carcinoma [3]. HNSCC, serving as the main subset of HNC, predominantly includes squamous cell carcinoma involving the oral cavity, pharynx and larynx [3]. Despite advances in surgery and chemoradiotherapy, the outcome improvement remains modest, with a 5-year survival rate lower than 50% [4]. Tumor invasiveness and metastasis, typical hallmarks of HNC, are largely responsible for the poor response to treatments [4, 5].

Metastasis is a common characteristic of cancer progression with multiple sequential steps, presenting as enhancement of the invasive ability of tumor cells and its spread to secondary sites of the body [6]. Epithelial-mesenchymal transition (EMT) represents a transcriptional program underpinning invasive and metastatic phenotype of cancers, which could be interpreted as a state of de-differentiation. Thus, EMT endows cancer cells with a high-grade phenotype to drive their migration, invasion and metastasis [7, 8]. Next, a subsequent step, the mesenchymal-epithelial transition, enables these spreading cells to colonize at a second location [9].

Long noncoding RNAs (lncRNAs) are one subtype of RNA transcripts which contain more than 200 nucleotides, lacking in capability of encoding protein or exhibiting limited potential [10]. LncRNA was previously considered as a genetic byproduct because of the absence of biological function [11]. Based on the upgraded DNA sequencing technologies, although the whole human genome is generally transcribed, more than 98% are non-encoding genes. Thus, it causes our conceptual shift regarding the possible role of the non-coding genes [12]. Besides, the importance of lncRNA in cancer biology is becoming appreciated by scholars. In recent years, based on high-throughput sequencing and biological techniques, increasing numbers of lncRNAs are being uncovered and their critical roles in regulating cancer development and progression are being extensively investigated. To date, the dysregulated expression and involvement of lncRNAs have been reported in diverse cancers, including HNC [13], lung cancer [14], breast cancer [15], colorectal cancer (CRC) [16], and esophageal squamous cell carcinoma (ESCC) [17]. Among these studies, lncRNAs seem to be implicated in each process of cancer progression, such as tumor development and metastasis. During metastasis, various lncRNAs are reported to function similarly or differently via diverse molecular mechanisms. Metastasis Associated Lung Adenocarcinoma Transcript 1 (MALAT1), as one lncRNA, was first detected and highly expressed within non-small cell lung cancer (NSCLC). Guo et al. demonstrated that MALAT1 exacerbates cell migration and invasion of NSCLC via binding to its downstream C-X-C motif chemokine ligand 5 (CXCL5). Additionally, the low methylated forms of the MALAT1 promoter in NSCLC accounts for its high expression [14]. Furthermore, homeobox transcript antisense RNA (HOTAIR) was indicated to potentiate the metastatic ability of colon cancer by inducing EMT [18]. In addition, increasing evidence has demonstrated that many lncRNAs could exert their biological function by working as pairs together with their adjacent mRNAs. Pan et al. indicated that the lncRNA Fork head box C1 upstream transcript (FOXCUT) could function together with Fork head box C1 (FOXC1) to potentiate the invasive and migrating capability of ESCC [17]. More importantly, the function and molecular mechanisms of these lncRNAs—i.e. MALAT1 [2], HOTAIR [19] and FOXCUT [20]—have been widely studied regarding their correlation with HNC metastasis. Given the important regulatory role of lncRNA in HNC metastasis, it is promising to be exploited as a prognosticator and therapeutic target for HNC.

In the present paper, we review these aberrantly expressing lncRNAs in the mediation of EMT, migration, invasion and metastasis of HNC (Table 1), and provide insight into their regulatory mechanism.

**Table 1:** LncRNAs implicated in EMT, invasion, migration and metastasis of HNC

LncRNA	Description	Cancer type	Expression changes in cancer	Correlation with tumor metastasis	Functions in HNC	Known molecular mechanisms	Ref.
AFAP1-AS1	Actin filament associated protein 1 antisense RNA1	NPC	up	positive	↑invasion, migration, metastasis	Translational control/ regulation of actin filament integrity	[69]
CCAT1	Colon cancer-associated transcript-1	LSCC	up	–	↑invasion	miRNA sponge/transcriptional regulation	[92]
CCAT1	Colon cancer-associated transcript-1	LSCC	up	–	↑invasion, migration, EMT	–	[91]
ENST00000470135	ENST00000470135	NPC	up	positive	↑invasion, migration	–	[73]
FOXCUT	FOXC1 promoter upstream transcript	NPC	up	–	↑migration	mRNA stability modulation	[20]
FOXCUT	FOXC1 promoter upstream transcript	OSCC	up	–	↑migration	mRNA stability modulation	[84]
GAS5	Growth Arrest-specific Transcript 5	TC	down	negative	–	–	[83]
H19	H19	LSCC	up	–	↑invasion, migration	Chromatin modification	[97]
HIT000218960	HIT000218960	PTC	up	positive	↑invasion, migration	Transcriptional regulation	[70]
HNF1A-AS	Hepatocyte nuclear factor 1A antisense RNA	NPC	up	–	↑EMT, migration	–	[107]
HOTAIR	Homeobox transcript antisense RNA	HNSCC	up	–	↑invasion, migration	miRNA sponge/translational control	[47]
HOTAIR	Homeobox transcript antisense RNA	OSCC	up	positive	↑invasion, migration	Chromatin modification	[19]
HOTAIR	Homeobox transcript antisense RNA	NPC	up	positive	↑invasion, migration	–	[48]
HOTAIR	Homeobox transcript antisense RNA	LSCC	up	–	↑invasion	Chromatin modification	[46]
HOTTIP	HOXA transcript at the distal tip	TSCC	up	positive	–	–	[76]
KCTD6–3	KCTD6–3	HNSCC	down	–	↓migration, EMT	–	[108]
LCE5A-1	LCE5A-1	HNSCC	down	–	↓migration, EMT	–	[108]
LINC00152	Long intergenic non-coding RNA 152	TSCC	up	positive	–	–	[77]
LINC00312	Long intergenic non-coding RNA 312	NPC	down	positive	–	–	[74]
LINC00312	Long intergenic non-coding RNA 312	NPC	up	–	↑invasion	Translational control/upregulation of JNK2/AP-1/MMP1 pathways	[75]
LINC00673	Long intergenic non-coding RNA 673	TSCC	up	–	↑invasion, migration	–	[105]
MALAT1	Metastasis Associated Lung Adenocarcinoma Transcript 1	OSCC	up	–	↑invasion, migration	Transcriptional regulation/NF-κB pathway activation	[2]
MALAT1	Metastasis Associated Lung Adenocarcinoma Transcript 1	TSCC	up	–	↑invasion, migration	–	[35]
MALAT1	Metastasis Associated Lung Adenocarcinoma Transcript 1	TC	up	–	↑invasion	Chromatin modification	[38]
MALAT1	Metastasis Associated Lung Adenocarcinoma Transcript 1	NPC	up	–	↑invasion, migration, EMT	–	[37]
MALAT1	Metastasis Associated Lung Adenocarcinoma Transcript 1	PTC	up	–	↑EMT	–	[39]
MALAT1	Metastasis Associated Lung Adenocarcinoma Transcript 1	TSCC	up	positive	↑EMT, migration, invasion	Chromatin modification/Wnt/β-catenin pathway activation	[34]
MALAT1	Metastasis Associated Lung Adenocarcinoma Transcript 1	TSCC	up	positive	↑migration, metastasis	Via downregulation of small proline rich proteins	[36]
NEAT1	Nuclear enrich abundant transcript 1	TC	up	–	↑invasion, migration	miRNA sponge	[102]
NKILA	NF-KappaB interacting lncRNA	TSCC	down	negative	↓EMT, migration, invasion, metastasis	Protein stability	[81]
NONHSAT037832	NONHSAT037832	PTC	down	negative	–	–	[82]
LncRNA-ROR	Long Non-Coding RNA Reprogramming	NPC	up	–	↑invasion, migration,EMT	–	[103]
RP11-169D4.1–001	RP11-169D4.1–001	LSCC	up	positive	–	–	[78]
SOX21-AS1	SOX21 antisense RNA 1	OSCC	down	–	↓invasion	Chromatin modification	[109]
TINCR	Terminal differentiation-induced ncRNA	HNSCC	down	–	↓migration	–	[110]
TUG1	Taurine upregulated gene 1	OSCC	up	positive	↑invasion	Transcriptional regulation/Wnt/β-catenin pathway activation	[66]
UCA1	Urothelial carcinoma-associated 1	HSCC	up	positive	↑invasion	–	[58]
UCA1	Urothelial carcinoma-associated 1	OSCC	up	positive	↑migration,invasion	Transcriptional regulation/Wnt/β-catenin pathway activation	[49]
UCA1	Urothelial carcinoma-associated 1	TSCC	up	positive	↑migration	–	[57]

*LncRNA* long noncoding RNA, *EMT* epithelial-mesenchymal transition, *HNC* head and neck cancer, *Ref.* reference, *NPC* nasopharyngeal carcinoma, *LSCC* laryngeal squamous cell carcinoma, *OSCC* oral squamous cell carcinoma, *TC* thyroid carcinoma, *PTC* papillary thyroid carcinoma, *HNSCC* head and neck squamous cell carcinoma, *TSCC* tongue squamous cell carcinoma, *HSCC* hypopharyngeal squamous cell carcinoma

## Functional mechanisms of lncRNAs in head and neck cancer

**Fig. 1:**
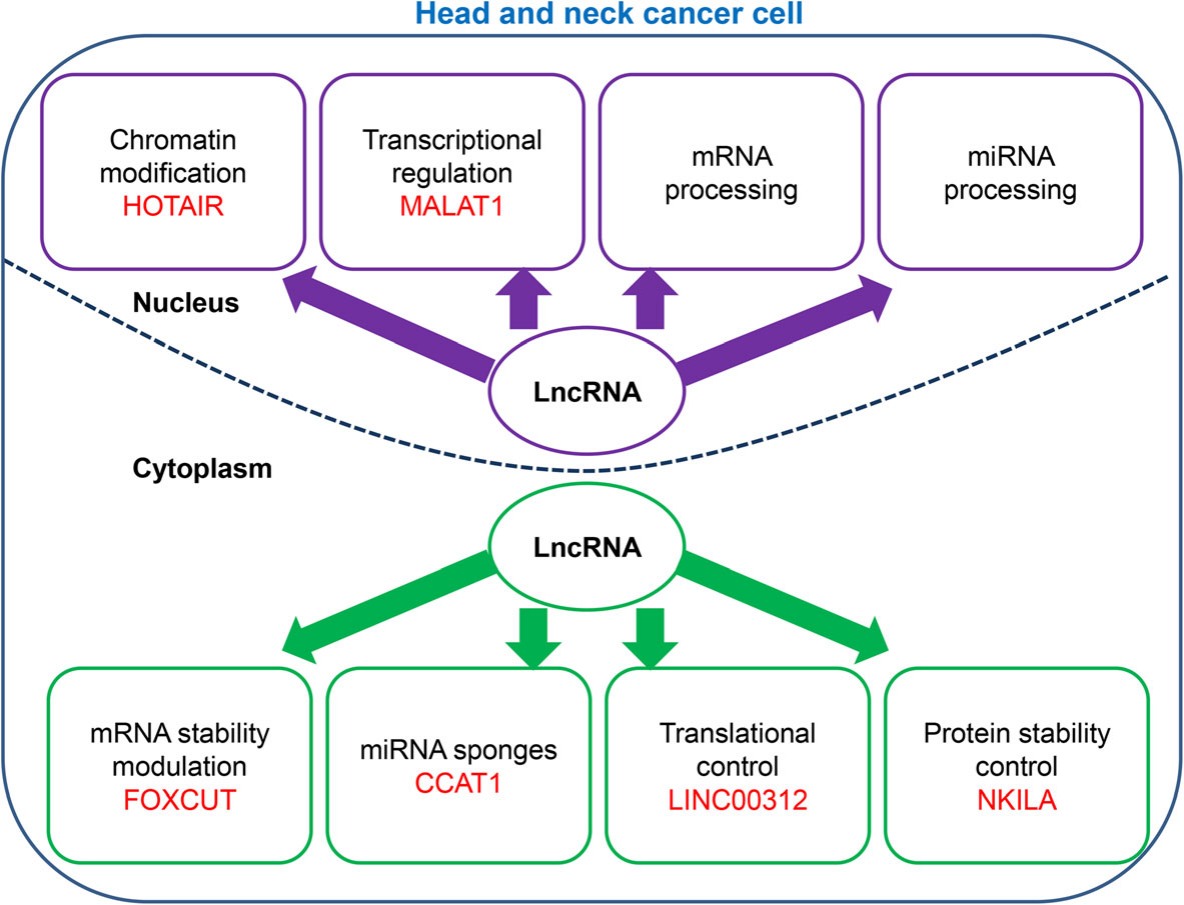
General mechanisms of HNC metastasis regulated by lncRNA based on their subcellular localization. Nulclear lncRNAs modulate metastasis-associated gene expression through chromatin modification, transcriptional control and mRNA/miRNA processing. For instance, mRNA processing includes alternative splicing of mRNA; besides, some lncRNAs could be spliced into pri-miRNAs, thus producing miRNAs. In the cytoplasm, lncRNAs serve as players in mRNA stability modulation, protein stability control, translational control and miRNA sponging. These lncRNAs with various functions in the HNC cell are highlighted in red as examples, while it is temporarily lacking in representatives for lncRNAs participating in HNC metastasis via mRNA/miRNA processing. LncRNA: long noncoding RNA; HNC: head and neck cancer; miRNA: microRNA

In general, lncRNAs perform their function based on their functional domains in the secondary or tertiary structure, and these mature structures originate from alternative splicing. Specifically, the domains facilitate the interaction of lncRNAs with chromatin, RNA and proteins; thus lncRNAs could function in HNC metastasis via chromatin remodeling, transcriptional control and post-transcriptional regulation [21, 22]. In general, lncRNAs could exist in the nucleus or cytoplasm, and different subcellular locations might determine the various biological functions of lncRNAs (Fig. 1) [23]. Therefore, lncRNAs are categorized into nuclear or cytoplasmic lncRNAs.

Several studies have suggested that most lncRNAs in the nucleus could function as a guide to lead chromatin modulating complexes into certain genomic loci and then initiate chromatin modification to activate or silence gene expression [24]. Additionally, nuclear lncRNAs could regulate gene transcription by their assembly with transcription factors into complexes. Also, they could be involved in the mRNA/miRNA processing; despite the absence of examples in HNC, some lncRNAs have been suggested to be implicated in mRNA/miRNA processing among other cancers [25, 26]. For instance, lncRNA colon cancer-associated transcript-2 (CCAT2), elevated in CRC, might repress the processing and maturation process of miR-145 in the nucleus, thereby restraining the proliferation and differentiation of CRC stem cells [25]; Additionally, as another lncRNA implied to interact directly with the serine/arginine splicing factor 1 in HeLa cells and influence the distribution and phosphorylation of the splicing factor, MALAT1 could potentially modulate the alternative splicing of pre-mRNAs, thus being involved in mRNA processing [26].

Regarding the great number of cytoplasmic lncRNAs, they typically modulate gene expression by base pairing with specific genes, improving or attenuating the mRNA stability and acting as miRNA sponges; in addition, they could influence mRNA translation by binding to different elements. Moreover, cytoplasmic lncRNAs are also responsible for protein stability control [24, 27].

## Overview of lncRNAs implicated in HNC metastasis

### LncRNAs that function positively in the metastasis of head and neck cancer

Presently, various lncRNAs have been demonstrated to function positively in metastasis, as well as in the EMT, migration and invasion of HNC. Here, we discuss their possible roles and attempt to elucidate the mechanisms.

#### MALAT1

MALAT1 was first uncovered as a poor prognosticator in NSCLC patients, whose overexpression could predict a higher risk of distant metastasis [28]. MALAT1 is an evolutionarily conserved and abundant lncRNA located in nuclear speckles, which are frequently implicated in epigenetic modulation and alternative splicing and drives the expression of metastasis-associated genes [29]. In the recent decade, numerous studies have revealed the potential of MALAT1 in modulating the invasion, migration and metastasis of various cancers, including ESCC, breast cancer, CRC and osteosarcoma [30–33]. Specifically, MALAT1 was indicated to be associated with the lymph node metastasis (LNM) of ESCC, contributing to its migratory and invasive abilities [30]; likewise, it promotes the metastasis of osteosarcoma in vitro and *vivo* through the phosphatidylinositol 3-kinase-protein kinase B (PI3K-AKT) pathway [31]. Additionally, Xu et al. indicated that one region of MALAT1 (from 6918 nt to 8441 nt) exhibits pivotal biological function in the migration and invasion of CRC [32]. However, MALAT1 was demonstrated to suppress the EMT of breast cancer through inhibiting the PI3K-AKT pathway [33]. In addition to the aforementioned phenotypes, MALAT1 has now been reported as a player in regulating the metastatic ability of HNC [2, 34–39].

Zhou and the colleagues, by employing siRNA to inhibit MALAT1 in HNC, indicated that the EMT, invasion and migration of HNC cells were attenuated possibly by inactivation of the β-catenin and nuclear factor-κB (NF-κB) pathways, which are potent regulators of EMT; furthermore, the in vivo study demonstrated the suppression of EMT markers, N-cadherin and Vimentin, in MALAT1 knockdown HNC tumors [2]. Moreover, another investigation achieved a similar conclusion. Upon tissue analysis, the positive correlation of MALAT1 with regional LNM of tongue squamous cell carcinoma (TSCC) patients was firstly validated. Subsequently, MALAT1, via modulation of the Wnt/β-catenin pathway, could induce the EMT, invasion and migration of TSCC; in turn, the MALAT1 impact was reversed by inhibiting the pathway, suggesting which as a key point to mediate the MALAT1 effect in TSCC [34]. Additionally, another study revealed the oncogenic role of MALAT1 in promoting TSCC metastasis in vivo [35]. Fang et al., with the same in vivo result, showed that MALAT1 knockdown might impair the migration of TSCC. DNA microarray results illustrated the upregulation of numerous small proline-rich protein (SPRR) family members after decreasing MALAT1, particularly the mRNA and protein levels of SPRR2A and SPRR2B. Additionally, an animal study showed that overexpressing SPRR2A could impair distant metastasis, implying the pivotal role of SPRR2A in MALAT-1-mediated metastasis of TSCC [36]. However, the underlying mechanism regarding how MALAT-1 targets SPRR2A awaits to be extensively studied.

Apart from HNSCC, MALAT1 also presents as a modulator of metastatic capability for other types of HNC in some in vitro studies. NPC is a disease more prevalent in southeastern Asia; despite the improvement in treatment modalities, distant metastasis remains a main culprit for its poor prognosis [37]. Xie and colleagues found that MALAT1 could potentiate the migration and invasion of NPC with upregulated EMT markers, namely, E-cadherin and vimentin [37]. One study proposed that MALAT-1 overexpression could upregulate the expression of the IQ-domain GTPase-activating protein 1 (IQGAP1), thereby increasing the invasion of thyroid carcinoma cells. Further investigation that IQGAP1 knockdown could reverse its invasion phenotype pinpoints IQGAP1 as a downstream target of MALAT1 [38]. However, another study suggested that, while MALAT1 promotes the EMT as well as metastasis of papillary thyroid carcinoma (PTC), it is markedly reduced in poorly differentiated thyroid carcinoma as well as anaplastic thyroid carcinoma, shedding light on the suppressor role of MALAT1 within other histological types of thyroid carcinoma [39].

#### HOTAIR

HOTAIR is located antisense to HOXC mRNA [40]. Mechanistically, HOTAIR was mainly demonstrated to function as a scaffold to induce epigenetic alteration. Specifically, the 5′ region of HOTAIR combines with the polycomb repressive complex 2 (PRC2), then redirects it into certain genomic sites; meanwhile, the 3′ region of HOTAIR might interact with lysine-specific demethylase 1 (LSD1). Next, HOTAIR coordinates these two major histone modification complexes to interplay with chromatin and consequently impairs anti-metastatic gene transcription [40, 41]. Mediated by PRC2, some findings have shown that HOTAIR enhances the metastasis of gastric, colorectal and breast cancer by targeting diverse downstream genes; in addition, HOTAIR impairs the migrating and invasive abilities of hepatocellular carcinoma cells by targeting RNA binding protein 38 [42–45].

Recently, HOTAIR was implicated to undertake a crucial role in HNC metastasis. Wu et al., after initially validating the positive association between HOTAIR expression and the LNM of oral squamous cell carcinoma (OSCC) within clinical samples, conducted an in vitro study and determined the oncogenic function of HOTAIR in driving the invasion and migration of OSCC. Their further data suggested that HOTAIR contributed to EMT by decreasing E-cadherin, the speculated mechanism of which is that HOTAIR could regulate the binding of the enhancer of zeste homolog 2 (EZH2), as well as trimethylation of lysine 27 in histone 3 (H3K27me3) to the promoter region of E-cadherin, thus enhancing the metastatic ability [19]. Moreover, a study regarding laryngeal squamous cell carcinoma (LSCC) demonstrated that HOTAIR knockdown significantly decreases the invasive ability of cancer cells. Further data implied that HOTAIR knockdown could attenuate the methylation level of phosphatase and tensin homolog deleted on chromosome ten (PTEN) via epigenetic modification, revealing a novel mechanism by which HOTAIR regulates LSCC invasion [46]. In addition, to identify the key factors in the HOTAIR regulatory circuit, Xu et al. suggested that HuR, an RNA binding protein, and HOTAIR might constitute a regulatory loop in driving the metastasis of HNC. In detail, they illustrated that HuR could interact with and stabilize HOTAIR, thereby promoting HOTAIR expression; in turn, HOTAIR positively improved the HuR level by acting as a miR-7 sponge, and HuR could also reinforce the sponge activity of HOTAIR [47]. Additionally, HOTAIR was clarified to positively correlate with the LNM of NPC, and the in vitro study proves that HOTAIR facilitates the migration and invasion in the NPC model [48].

#### UCA1

Urothelial carcinoma-associated 1 (UCA1) comprises of three exons and encodes two transcripts [49]. lncRNA UCA1 was initially detected to be significantly upregulated in bladder transitional cell carcinoma [50]. It has two isoforms, which are 1.4 kb and 2.2 kb in length, respectively. Additionally, the alignment of DNA sequences showed that these two isoforms share 1265 bp of the common region [51, 52]. Until now, several groups have highlighted the metastasis-modulating effect of UCA1 in various cancers [53–56]. Zuo uncovered that the elevation of UCA1 is correlated with the LNM of gastric cancer (GC); besides, UCA1 regulates the EMT of GC cells possibly through transforming growth factor-β1 (TGFβ1) induction in vitro [53]. After observing the positive correlation of UCA1 with the LNM of endometrial cancer, scholars have confirmed that the deficiency in UCA1 could reduce migration and invasion [54]. Aside from that, UCA1 was indicated as a sponge for miR-485-5p in ovarian cancer, thus increasing matrix metallopeptidase 14 (MMP14) expression and enhancing the metastasis [55]. Beyond that, UCA1 silencing attenuates the migrating ability of melanoma cells [56].

Recently, a few studies have unveiled the function of UCA1 in HNC metastasis. As a lncRNA dysregulated in TSCC, UCA1 is closely related to its LNM, and its silencing markedly dampens cancer invasiveness. Further study demonstrated that UCA1 triggers Wnt/ β-catenin pathway activation and contributes to cancer metastasis [49]. Similarly, Fang et al. reported that UCA1 is not only correlated with the LNM of TSCC, but could also enhance the migration of TSCC cells [57]. Apart from OSCC, UCA1 also plays a pivotal role in hypopharyngeal squamous cell carcinoma; with the initial trial confirming its positive correlation with the LNM of hypopharyngeal squamous cell carcinoma, the UCA1 loss-of-function test in Fadu cells validated its role in driving invasion [58], whereas further mechanistic study is required to elucidate the phenotype.

#### TUG1

Taurine upregulated gene 1 (TUG1) is another frequently reported lncRNA [59]. Previously, it was found to be dysregulated within several cancers. In CRC, both the gain- and loss-of-function tests proved the positive impact of TUG1 on improving the invasion and migration of tumor cells via regulating EMT-related proteins. In addition, the overexpression of TUG1 promotes the liver metastasis of CRC [60]. Likewise, it was similarly observed to boost the metastatic capacity of renal cell carcinoma, gallbladder carcinoma, ESCC and ovarian cancer [61–64]. Recently, a meta-analysis indicated that TUG1 was positively associated with the LNM of several cancers [65].

To date, only one study has discussed TUG1 in HNC metastasis. Liang and colleagues, after confirming the positive correlation between TUG1 and LNM, showed that TUG1 might inhibit the invasion of OSCC along with the downregulation of β-catenin, while a stimulator of the Wnt/β-catenin pathway could reverse the repression effect of TUG1 [66]. Thus, the pathway was postulated as how TUG1 regulates OSCC metastasis. Extensive studies are desired to uncover its underlying functions and mechanisms for mediating HNC metastasis.

#### AFAP1-AS1

Actin filament associated protein 1 antisense RNA 1 (AFAP1-AS1), which is derived from the complementary chain of the Actin Filament Associated Protein 1 (AFAP1) gene, is presently implicated in modulating the metastasis of lung cancer, hepatocellular carcinoma, and notably, NPC [67–69]. Initially, AFAP1-AS1 was positively associated with the LNM and distant metastasis of NPC in tissue verification. Further in vitro and in vivo studies consolidated its function in facilitating NPC metastasis. Mechanistically, AFAP1-AS1 might exert its effect on NPC metastasis via maintaining the actin filament integrity [69], with the same mechanism validated in lung cancer [67].

#### HIT000218960

After investigating the lncRNAs profiles between PTC tissue and normal thyroid tissue, Li et al. identified an obviously elevated lncRNA in PTC, termed as HIT000218960, which is related to LNM of PTC. In addition, inhibition of HIT000218960 repressed migration and invasion of PTC possibly by downregulating high mobility group AT-hook 2 (HMGA2) mRNA level [70]. However, the mechanism is still elusive. Based on previous studies, two other lncRNAs have functioned as endogenous competitors with miRNAs targeting HMGA2 [71, 72], which is speculated to play an identical role as HIT000218960. Considering the other lncRNAs as a reference, the detailed mechanism through which HMGA2 is regulated by HIT000218960 is anticipated to be illuminated in the future.

#### Other potential pro-metastasis lncRNAs in HNC

ENST00000470135 is a lncRNA identified to be markedly elevated in highly metastatic NPC cells, and its positive role in the LNM of NPC was further confirmed. Additionally, knocking down ENST00000470135 could remarkably repress the migration and invasion of NPC cells [73]. As another representative, long intergenic non-coding RNA 312 (LINC00312), known as NPC-associated gene 7 (NAG7), was also positively correlated with the LNM of NPC based on a tissue study [74]. Furthermore, the overexpression of LINC00312 potentiates NPC invasion by repressing estrogen receptor α and stimulating the c-Jun N-terminal kinase-2/activator protein-1/matrix metalloproteinase-1 (JNK2/AP-1/MMP1) pathway [75].

HOXA transcript at the distal tip (HOTTIP), as a lncRNA highly expressed in HNC, is associated with its distant metastasis and is responsible for its dismal prognosis [76]. Relying on previously published data concerning TSCC gene expression profiling, Yu et al. discovered a novel lncRNA named long intergenic non-coding RNA 152 (LINC00152), which is enhanced in TSCC, and further tissue investigations consolidated its role in promoting the LNM of TSCC [77]. Additionally, a lncRNA microarray based on LSCC samples suggested the upregulation of RP11-169D4.1–001, which was subsequently observed to be positively associated with the LNM of LSCC [78]. Overall, these findings have uncovered several lncRNAs in HNC metastasis; however, their actual function and mechanisms need to be thoroughly elucidated, enabling which to become therapeutic targets of HNC.

### LncRNAs act as negative regulator in head and neck cancer metastasis

To date, only a few lncRNAs have been highlighted to suppress HNC metastasis. Herein, we generally introduced them and investigated NKILA as a representative.

#### NKILA

Nuclear factor-κB interacting lncRNA (NKILA) is major inhibiting checkpoint for NF-κB activation in breast cancer [79]. Encoded by a gene at chromosome 20q13, NKILA was first shown to exhibit an inhibitory effect on the metastatic abilities of breast cancer cells [79]. In light of the remarkable relationship between NF-κB and the invasiveness of tumor cells [80], Liu and colleagues attempted and successfully confirmed the putatively negative regulation of breast cancer metastasis by NKILA. Mechanistically, NKILA is mainly upregulated by NF-κB activation; in turn, NKILA forms a negative feedback loop to suppress NF-κB by binding the cytoplasmic NF-κB/IκB compound and forming another stable NF-κB:IκB:NKILA complex, and suppressing the phosphorylated level of IκB, thereby preventing the nuclear translocation of NF-κB and exerting its anti-metastasis impact on breast cancer. Moreover, in vivo and tumor sample studies confirmed its role as a negative regulator of metastasis and predictor of poor prognosis. Moreover, miR-103 and miR-107 were demonstrated to reverse the effect by degrading NKILA, thereby enabling NF-κB activation and promoting metastasis [79].

According to the previous finding about NKILA, the same group also reported its similar influence on TSCC metastasis. To begin with, NKILA was pervasively reduced in TSCC samples by contrast with that in normal tongue tissue; notably, it was negatively correlated with the LNM of TSCC. Cell invasion and migration studies further verified its negative role in TSCC. In theory, the mechanism of NKILA in downregulating the TSCC metastatic capacity is identical as to that in breast cancer. NKILA could initially bind to IκB, suppressing NF-κB activation and thereby blocking its downstream EMT phenotype. In agreement, animal investigations also showed that NKILA knockdown contributed to more lung metastasis of TSCC [81]. Overall, it implies that lncRNA might bind to the functional domain of vital signaling molecules and regulate the protein stability, thus affecting tumor metastasis.

## Other tumor metastasis-suppressing lncRNAs

Based on microarray analysis, NONHSAT037832 was detected as a novel lncRNA remarkably downregulated in PTC, next implied to function as an inhibitor in the LNM of PTC [82]. Besides, another study indicated that the expression level of lncRNA Growth Arrest-specific Transcript 5 (GAS5) was negatively related to the LNM of thyroid carcinoma [83]. Moreover, further studies are desired to determine their detailed mechanisms in attenuating tumor metastasis.

### LncRNAs serve as promoter in the EMT, migration and invasion of head and neck cancer

Presently, diverse lncRNAs are solely involved in driving the EMT, migration and invasion of HNC, whereas the in vivo functional roles await deep discovery.

#### FOXCUT

FOXCUT is a lncRNA encoded from the upstream area of the FOXC1 promoter. As indicated, FOXCUT functions together with FOXC1 as a lncRNA-mRNA pair in enhancing the migration of OSCC and NPC cells [20, 84]. Presently, various studies have indicated that lncRNAs could collaborate with mRNAs transcribed from their neighboring area as pairs and regulate their own function, opening a new perspective to study lncRNA function [85]. By bioinformatics analysis, FOXCUT was detected as an overexpressed lncRNA in OSCC, similar to that for FOXC1 mRNA. In addition, FOXC1 mRNA was remarkably reduced after the knockdown of FOXCUT, implying that FOXC1 expression is modulated by FOXCUT. Of note, the knockdown of FOXCUT or FOXC1 could suppress the migration of OSCC cells, possibly mediated by the reduction of the MMP2, MMP7, and MMP9 levels [84]. Analogously, FOXCUT and FOXC1 are both overexpressed in NPC cells and tissues, and tissue validation confirmed their synergistic effect on promoting the distant metastasis of NPC. An in vitro study suggested that the FOXCUT-FOXC1 pair interacted with each other, and the silencing of FOXCUT inhibited the migration of NPC cells, along with the decrease in the MMP7, MMP9 and β-catenin levels [20]. Notably, the same effect of this pair was confirmed in the basal-like breast cancer study [86]. Nevertheless, whether this pair could regulate the invasion and metastasis of cancer, as well as its underlying mechanisms, are required to be thoroughly investigated in the future.

#### CCAT1

Colon cancer-associated transcript1 (CCAT1) serves as a lncRNA involved in regulating the metastatic ability of various cancers via distinct or similar mechanisms, including colon cancer [87], cervical cancer [88], hepatocellular carcinoma [89] and melanoma [90] etc. Zhuang et al. first uncovered the metastasis-related functions of CCAT1 in HNC. The study suggests that CCAT1 could improve the migrating and invasive abilities of LSCC cells by leading to EMT, presenting as E-cadherin reduction along with the enhancement of Vimentin and N-cadherin [91]. On the other hand, a CCAT1/ miR-218/zinc finger protein, x-linked (ZFX) axis was identified to significantly modulate the invasion of LSCC. Through tissue investigation, CCAT1 and ZFX were confirmed to be significantly enhanced, while miR-218 was decreased. The gain or loss of function study ultimately revealed that CCAT1 drives the invasion of LSCC cells through increasing the ZFX level by sponging miR-218 [92]. All the above imply that the same lncRNA might regulate metastasis via diverse mechanisms.

#### H19

lncRNA H19 is the transcript product of the H19 gene [93]. Emerging findings have supported its pivotal role as a metastatic potentiator in various malignancies, including GC [94], breast cancer [95] and colon cancer [96]. In GC cells, lncRNA H19 could be processed into miR-675, which subsequently activates protein kinase B/mammalian target of rapamycin (Akt/mTOR) pathway as well as enhances the invasion of GC cells; therefore, the function of H19 is partially dependent on its downstream product miR-675 [94]. More importantly, the investigation has demonstrated the influence of H19 silencing on impairing the migration and invasion of LSCC; specifically, H19 might sponge miR-148a-3p, thereby releasing the DNA methyltransferase enzyme (DNMT1) which could be targeted by miR-148a-3p, and carrying out its function [97]. Thus, the lncRNA H19/miR-148a-3p/DNMT1 axis presents as a vital signaling cascade that mediates LSCC metastasis. Since H19 has various effects on other maliganancies, its function in HNC requires extensive investigation.

#### NEAT1

Nuclear enriched abundant transcript 1 (NEAT1), as its name implies, is a lncRNA localized exclusively to paraspeckles, a sub-nuclear structure [98]. Previous studies have identified the potential of lncRNA NEAT1 in enhancing the invasion and migration of GC via driving EMT [99]. Additionally, upon analyzing the relationship between expression of NEAT1 and the LNM of NSCLC [100] and CRC [101], NEAT1 was reported to significantly promote their metastasis, proving its oncogenic role. Notably, NEAT1 acts a promoting role in mediating the migration and invasion of thyroid carcinoma. Specifically, NEAT1, by exhibiting a reciprocal repression correlation with miR-214 and reducing its expression, increases the β-catenin level and presents the phenotype [102].

#### lncRNA-ROR

LncRNA-Regulator of Reprogramming (LncRNA-ROR) is overexpressed in solid tumors including NPC. LncRNA-ROR deficiency contributes to the suppressed tumor invasion with reduced expression levels of the EMT marker [103]. Similarly, LncRNA-ROR also potently drives the invasion and lung metastasis of breast cancer through EMT [104].

Emerging lncRNAs are also implicated in facilitating HNC metastasis. For example, long intergenic non-coding RNA 673 (LINC00673) was identified to be the most highly expressed lncRNA in TSCC based on the microarray analysis of two TSCC cohorts in the Gene Expression Omnibus dataset. Thereafter, it was validated in TSCC samples [105]. Additionally, silencing of LINC00673 could remarkably repress the invasive and migratory abilities of TSCC, similar to another finding in a GC study [106]. Hepatocyte nuclear factor 1A antisense RNA (HNF1A-AS) is another lncRNA detected to be elevated in NPC, the knockdown of which could impair the migration of NPC cells [107].

### LncRNAs that are inversely correlated with the EMT, migration and invasion of HNC

Apart from these lncRNAs that are positively related to the metastatic ability of HNC in vitro, a few lncRNAs are inversely correlated with its metastasis in vitro. Zou et al. carried out transcriptome sequencing to investigate dysregulated lncRNAs in HNC; as a result, they discovered two dramatically reduced lncRNAs, namely, KCTD6–3 and LCE5A-1. Moreover, transfection of both lncRNAs into HNC cells reduced its migration significantly because these two lncRNAs might lead to EMT alterations. Specifically, KCTD6–3 could decrease vimentin and twist, and LCE5A-1 might increase E-cadherin, while reducing vimentin [108]. In another study concerning OSCC, investigators used next-generation sequencing approach to analyze transcriptome profiling and identified that lncRNA SOX21 antisense RNA 1 (SOX21-AS1) was significantly decreased, mainly owing to aberrant promoter hypermethylation of SOX21-AS1. Furthermore, SOX21-AS1 overexpression could remarkably inhibit the invasion of OSCC cells. Additionally, subcellular fractionation localization implies that it might serve as a suppressor for tumor metastasis in the nucleus, while the precise mechanisms need to be illuminated [109]. In addition, terminal differentiation-induced ncRNA (TINCR), as a lncRNA upregulated by zinc-finger 750 (ZNF750) in HNC cells, could promote its migration [110]. Of note, the detailed ration regarding how these lncRNAs inhibit EMT, migration or invasion in vitro is worthy of identification, thus facilitating their application as prognostic predictors.

## LncRNAs regulate tumor metastasis by interacting with tumor immunity

**Fig. 2:**
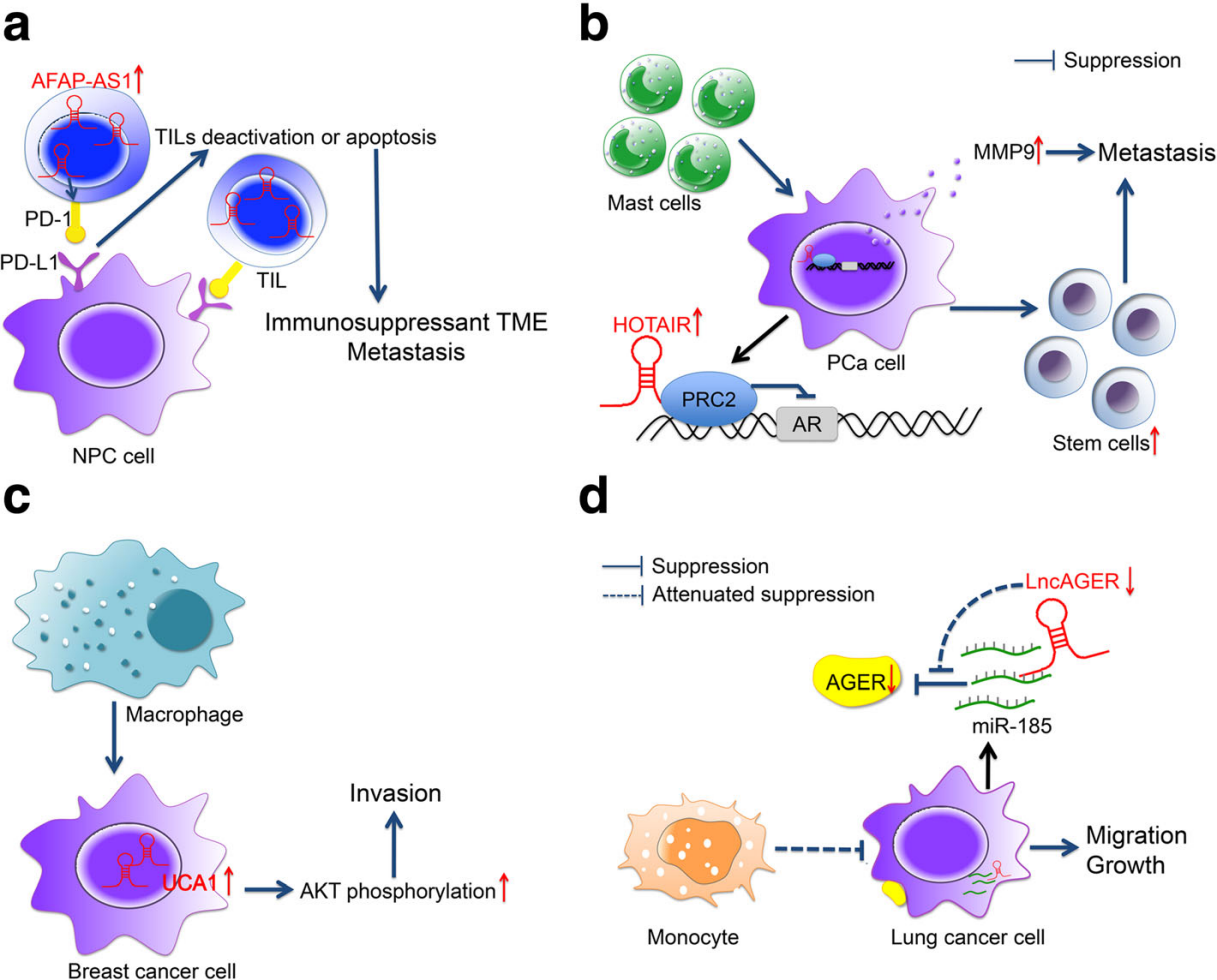
LncRNAs potentially modulate tumor metastasis through interacting with tumor immunity. **a** AFAP1-AS1 might contribute to the apoptosis or deactivation of TILs by increasing PD-1 expression in TILs, which is an immune escape marker, thus leading to immunosuppressant TME and metastasis of NPC. **b** HOTAIR could integrate with PRC2 as a complex under stimulation of mast cells to suppress AR, and thus increase MMP9 levels and the stem/progenitor cell population, contributing to the metastasis of prostate cancer cells. **c** Infiltrating macrophages in TME could potentiate invasion of breast cancer in vitro by increasing AKT phosphorylation, thus boosting level of lncRNA UCA1. **d** LncAGER could attenuate the tumor migration and growth of lung cancer via targeting miR-185, thus reversing the impact of miR-185 on inhibiting AGER expression in lung cancer cells and inducing the anti-tumor effect of human monocytes. lncRNAs: Long noncoding RNAs; AFAP1-AS1: Actin filament associated protein 1 antisense RNA 1; TILs: Tumor infiltrating lymphocytes; PD-1: Programmed death 1; TME: Tumor microenvironment; NPC: Nasopharyngeal carcinoma; HOTAIR: Homeobox transcript antisense RNA; PRC2: Polycomb repressive complex 2; AR: androgen receptor; PCa: prostate cancer; UCA1: Urothelial carcinoma-associated 1; AKT: Protein Kinase B; AGER: advanced glycosylation end-product specific receptor; lncAGER: lncRNA AGER; miR-185: microRNA-185

The tumor microenvironment (TME) of HNC is characterized by immunosuppression [111]. CD8+ T cells among the tumor-infiltrating lymphocytes (TILs) express significantly higher levels of immune checkpoint receptors such as programmed cell death 1 (PD-1) than those in the peripheral blood [112, 113]. Interestingly, lncRNAs can directly regulate the expression levels of the checkpoint receptors and their ligands (Fig. 2a) [114]. For example, the lncRNA AFAP1-AS1 upregulates PD-1 expression levels in the TME of NPC, possibly leading to T-cell exhaustion. [114]. TME exhibits strong M2-like skewing, which dampens antigen-presenting cell function and subsequent tumor-specific effector activation. Some TIL subsets such as mast cells appear to contribute to immunosuppression and enhance cancer invasion. Notably, mast cells could directly increase the expression of HOTAIR and lead the HOTAIR-PRC2 complexes to suppress the expression of androgen receptor, thereby contributing to tumor invasion (Fig. 2b) [115]. As another lncRNA exacerbating the M2-like phenotype in the TME, tumor-associated macrophages could potentiate the invasion of breast cancer in vitro by upregulating UCA1 (Fig. 2c) [116]. By contrast, lncRNA advanced glycosylation end-product specific receptor (lncAGER) could strengthen the effect of human monocytes against lung cancer, thereby inhibiting tumor migration and growth (Fig. 2d). Although downregulated in lung cancer due to the hypermethylation of its promoter, lncAGER exhibits the anti-tumor function by first targeting miR-185, thereby increasing the advanced glycosylation end-product specific receptor (AGER) level in lung cancer cells, which is an important innate immune pattern-recognition receptor, and ultimately promoting the anti-tumor effect of human monocytes [117]. Overall, how lncRNAs in cancer cells regulate the immune microenvironment of HNC remains insufficiently characterized. However, emerging evidence suggests that targeting a subset of lncRNAs not only alleviates the cancer invasion phenotype but also potentially contributes to improved immune detection of cancer.

## Upstream regulator for the aberrant expression of LncRNAs in HNC

Based on previous findings, there are three main mechanisms that are responsible for the aberrant expression of lncRNA in HNC, which are miRNAs, functional proteins such as RNA binding proteins (RBPs), NF-κB and TP53, and genetic changes such as genomic mutation and epigenetic alteration [47, 81, 118–125]. Primarily, there are two miRNAs that modulate lncRNA expression in HNC. Specifically, lncRNA papillary thyroid carcinoma susceptibility candidate 3 (PTCSC3) could be remarkably decreased by the overexpression of miR-574-5p in thyroid carcinoma [118]; additionally, in TSCC, miR-26a could upregulate the lncRNA maternally expressed gene 3 (MEG3) by binding to the DNA methyltransferase 3B transcript [119]. Second, two RBPs are involved in regulating lncRNA expression in HNC. HuR, as an RBP, could form a regulatory circuit with HOTAIR and contribute actively to the stability and expression of HOTAIR in HNC [47]; similarly, HuR could regulate the stability of lnc-Sox5 and lead to TSCC progression [120]; in addition, another RBP, RNA-binding protein 24 could degrade MALAT1 by directly upregulating miR-25 expression, revealing the synergistic effect of RBP and miRNA on regulating lncRNA in NPC [121]. NF-κB is another functional protein—i.e. in TSCC, NF-κB could significantly improve the expression level of lncRNA NKILA [81]; BamHI-A rightward transcripts (BARTs), including lncRNAs, are produced by Epstein-Barr virus (EBV) in NPC, and NF-κB functions positively to activate BART promoters and modulate the expression of these lncRNAs [122]. As another representative of functional protein, TP53 could directly upregulate lncRNA LOC401317 in NPC cells, thereby suppressing tumor growth [123].Third, genetic changes such as single-nucleotide polymorphisms (SNPs) or epigenetic alterations within the non-coding genome could markedly affect the transcription of lncRNA; for instance, in PTC, the polymorphism of rs944289 in 14q13.3 could reduce the level of lncRNA PTCSC3 by abolishing the binding domain of CCAAT/enhancer binding proteins α and β and subsequently inhibiting the activation of the PTCSC3 promoter [124]. Additionally, the silencing of lncRNA H19 in well-differentiated NPC cells is attributed to the epigenetic alteration, namely, hypermethylation of the H19 promoter region [125]. Overall, the aberrant expression of lncRNAs in HNC is mainly controlled by the above three upstream regulators, and more upstream modulators are required to be uncovered.

## Conclusions

In the past decade, lncRNAs, previously regarded as non-functional [11], have attracted considerable and increasing attention from investigators, primarily attributed to their frequently aberrant expression in cancers and their potential implication in tumor development and progression, particularly tumor metastasis [16], which constitutes the main threat for cancer-related death. LncRNAs, located in the nucleus or cytoplasm, might interact with mRNA, miRNA, DNA or protein to exert their diverse functions [21, 22], acting as a versatile player in regulating neoplasm metastasis.

In this review, we introduced the lncRNAs implicated in modulating the metastatic potential of HNC and attempted to illuminate the mechanisms; meanwhile, the involvement of these lncRNAs in the metastasis of other common malignancies are also briefly summarized to facilitate their study in HNC. Additionally, the upstream regulation of lncRNA that underlies its abnormal expression in HNC was discussed. Based on the screening result of lncRNAs, most of these are suggested to promote the EMT, migration, invasion or metastasis of HNC, while a small proportion of these have opposite effects of inhibiting HNC metastasis in vitro and/or in vivo. In theory, these lncRNAs highly expressed in HNC tissue or cells, driving its metastasis, have the potential to be utilized as therapeutic targets and predictors of poor outcome. However, the prerequisite of its application is the illumination of mechanisms validated in vitro and in vivo. Moreover, those lncRNAs downregulated in HNC and exhibiting the capability of suppressing its metastasis also hold the promise to serve as prognostic predictors.

In terms of clinical translation of lncRNAs, it still has a long way to go since there exist multiple unmet challenges for us to overcome. In order to realize the goal, we need to seek solutions for the listed issues: 1. exploring more lncRNAs affecting HNC metastasis; 2. investigating the detailed mechanisms of these lncRNAs; 3. figuring out the key lncRNAs pathways; 4. constructing a lncRNA interacting and regulating network since TME is complex and not dependent on a single lncRNA; 5. since HNC includes a range of cancers with various genetic background, individualized key lncRNAs and its involved pathways in a specific cancer might be studied; 6. intervening tumor metastasis-promoting lncRNAs to inhibit metastasis by targeting lncRNAs.

To target lncRNAs in HNC, diverse techniques have been developed, namely, RNA interference (RNAi) [126], antisense oligonucleotides(ASO) [127], clustered regularly interspaced short palindromic repeats (CRISPR)/Cas9 [128], RNA blocking oligonucleotides [129] and small-molecule modulators [130]. The RNAi technique represents selective silencing of lncRNAs, which is supposed to be more efficient in depleting cytoplasmic lncRNAs, while the successful rate of the deletion of nuclear lncRNAs remains unsatisfied [126, 131]. ASO is another effective approach to target lncRNAs regardless of the cellular location, which applies single-stranded DNA or RNA molecules to direct the RNase H to target lncRNAs, leading to its degradation [127]. One good example of its preclinical study proposed that ASO of MALAT1 could inhibit lung cancer metastasis in vivo significantly [132]. CRISPR/Cas9, as a relatively novel and efficient genome editing tool, has shed light on a novel way to edit lncRNA expression, while its specificity and efficacy remain to be further evaluated [27, 128]. As RNAi and ASO-mediated lncRNA degradation are dependent on enzymatic degradation, limiting the capability of improving their pharmacological qualities, RNA-blocking oligonucleotides is another method that modulates lncRNA by blocking the access of the cellular machinery to the RNA rather than contributing to the degradation of the lncRNA, which do not employ enzymes for the activity, exhibiting the advantage to receive more chemical modifications that enhance their drug-like characteristics [129]. In addition, small-molecule modulators involved in interrupting the lncRNA–protein interaction display high potential to target the lncRNA specifically, reducing its off-target effects [130]. Further validation of these above techniques and more technical innovations are required to select the optimal drug for lncRNA-targeted therapeutics, considering their advantages and disadvantages.

Recently, a novel approach, namely, clustered regularly interspaced short palindromic repeats (CRISPR)-Display, has emerged as a potentially transformative tool to probe into the function and mechanisms of lncRNAs [133]. Specifically, Shechner et al. established the technology by employing a nuclease-deficient Sp. dCas9 mutant, namely, “dCas9”, which displays certain RNA domains on the dCas9 cargo and is delivered to the predetermined DNA loci. Attempting to validate the applicability of the approach for lncRNAs, Shechner et al. fused the RepA domain of lncRNA Xist into the complexes; expectedly, they showed that the domain modestly repressed the expression of reporter gene, which initially revealed the plausibility of CRISPR-Display (CRISP-Disp) for studying lncRNAs [133]. In light of its characteristics, CRISP-Disp presents its potential advantages in lncRNA research. First, it could be utilized to address the question regarding whether lncRNA-regulated gene expression is due to the transcriptional effect or lncRNA domain itself [133]. Second, CRISP-Disp and immunoprecipitation of dCas9 followed by mass spectrometry could assist in identifying interacting proteins with the lncRNA domain [134]. Third, the approach might efficiently serve as a platform for selecting the novel functional domain of lncRNA. Fourth, the method could be applied to study lncRNA up to 4.8 kb and simultaneously explore multiple RNA domains by sharing the same dCas9 [133]. However, more validations are warranted to ensure its application in the future.

In summary, lncRNAs contribute to HNC invasion and metastasis by distinct mechanisms based on their subcellular localization, as well as modulating or regulated by the cancer immune microenvironment. Emerging evidence has identified lncRNAs as a novel set of potential prognosticators and therapeutic targets. The characterization of an expanded repertoire of lncRNAs and their interaction with the cancer immune microenvironment are promising to further improve combinatorial treatment protocols for HNC.

## Abbreviations

AFAP1: Actin Filament Associated Protein 1
AFAP1-AS1: Actin filament associated protein 1 antisense RNA 1
AGER: Advanced glycosylation end-product specific receptor
Akt/mTOR: Protein kinase B/mammalian target of rapamycin
ASO: Antisense oligonucleotides
BARTs: BamHI-A rightward transcripts
CCAT1: Colon cancer-associated transcript1
CCAT2: Colon cancer-associated transcript-2
CRC: Colorectal cancer
CRISP-Disp: CRISPR-Display
CRISPR: Clustered regularly interspaced short palindromic repeats
CXCL5: C-X-C motif chemokine ligand 5
DNMT1: DNA methyltransferase enzyme 1
EBV: Epstein-Barr virus
EMT: Epithelial-mesenchymal transition
ESCC: Esophageal squamous cell carcinoma
EZH2: Enhancer of zeste homolog 2
FOXC1: Fork head box C1
FOXCUT: FOXC1 upstream transcript
GAS5: Growth arrest-specific transcript 5
GC: Gastric cancer
H3K27me3: Trimethylation of lysine 27 in histone 3
HMGA2: high mobility group AT-hook 2
HNC: Head and neck cancer
HNF1A-AS: Hepatocyte nuclear factor 1A antisense RNA
HNSCC: Head and neck squamous cell carcinoma
HOTAIR: Homeobox transcript antisense RNA
HOTTIP: HOXA transcript at the distal tip
IQGAP1: IQ-domain GTPase-activating protein 1
JNK2/AP-1/MMP1: C-Jun N-terminal kinase-2/activator protein-1/matrix metalloproteinase-1
LINC00152: Long intergenic non-coding RNA 152
LINC00312: Long intergenic non-coding RNA 312
LINC00673: Long intergenic non-coding RNA 673
LncAGER: LncRNA advanced glycosylation end-product specific receptor
LncRNA-ROR: LncRNA-Regulator Of Reprogramming
lncRNAs: Long noncoding RNAs
LNM: Lymph node metastasis
LSCC: Laryngeal squamous cell carcinoma
LSD1: Lysine-specific demethylase 1
MALAT1: Metastasis associated lung adenocarcinoma transcript 1
MEG3: Maternally expressed gene 3
MMP14: Matrix metallopeptidase 14
NAG7: NPC-associated gene 7
NEAT1: Nuclear enriched abundant transcript 1
NF-κB: Nuclear Factor-κB
NKILA: Nuclear Factor-κB Interacting LncRNA
NPC: Nasopharyngeal carcinoma
NSCLC: Non-small-cell lung cancer
OSCC: Oral squamous cell carcinoma
PD-1: Programmed death 1
PI3K-AKT: Phosphatidylinositol 3-kinase-protein kinase B
PRC2: Polycomb repressive complex 2
PTC: Papillary thyroid carcinoma
PTCSC3: Papillary thyroid carcinoma susceptibility candidate 3
PTEN: Phosphatase and tensin homolog deleted on chromosome ten
RBPs: RNA binding proteins
RNAi: RNA intereference
SgRNA: Single guide RNA
SNPs: Single nucleotide polymorphisms
SOX21-AS1: SOX21 antisense RNA 1
SPRR: Small proline rich proteins
TGFβ1: Transforming growth factor-β1
TILs: Tumor infiltrating lymphocytes
TINCR: Terminal differentiation-induced ncRNA
TME: Tumor microenvironment
TSCC: Tongue squamous cell carcinoma
TUG1: Taurine upregulated gene 1
UCA1: Urothelial carcinoma-associated 1
ZFX: zinc finger protein, X-linked messenger RNA
ZNF750: Zinc-finger 750
